# Integrative Transcriptomic Analysis and Single-Cell Characterization Identify *RTN4* as a Candidate PBMC-Derived Hub Gene Associated with COPD and Coronary Artery Disease

**DOI:** 10.3390/genes17091046

**Published:** 2026-08-29

**Authors:** Yongle Xu, Shan Shan, Hanhan Liu, Tao Ren

**Affiliations:** Department of Respiratory and Critical Care Medicine, Shanghai Sixth People’s Hospital, Shanghai Jiao Tong University School of Medicine, Shanghai 200233, China; leyongx@126.com (Y.X.); shanshan_shcn@126.com (S.S.)

**Keywords:** chronic obstructive pulmonary disease, coronary artery disease, *RTN4*, monocytes, immunometabolic reprogramming

## Abstract

**Background:** Chronic obstructive pulmonary disease (COPD) is associated with systemic inflammation and increased cardiovascular comorbidity, yet peripheral blood molecular markers for cardiovascular comorbidity-related stratification in COPD remain poorly defined. **Methods:** In this study, PBMC transcriptomic datasets from a COPD cohort (GSE42057) and a CAD cohort (GSE113079) were analyzed using weighted gene co-expression network analysis (WGCNA) to identify disease-associated modules, followed by overlapping gene screening. Machine-learning models were then applied to prioritize the shared genes. External validation was performed in an independent COPD cohort (GSE54837) and an independent CAD cohort (GSE250283). Immune-cell deconvolution and single-cell transcriptomic analysis of a CAD dataset (GSE269269) were further used to characterize the immune and cellular context of the leading candidate. Finally, RT–qPCR was performed in an institutional PBMC cohort for experimental validation. **Results:** A total of 169 shared genes were identified, with enrichment in immune, mitochondrial, oxidative phosphorylation, and metabolic pathways. *RTN4* was the most consistently validated candidate across COPD and CAD cohorts and was associated with poorer lung function, advanced GOLD stages, and monocyte-related immune patterns. In CAD single-cell data, *RTN4*-associated signals were mainly localized to monocytes, particularly intermediate monocytes under plaque rupture conditions, with enrichment of immune, antigen-presentation, oxidative-stress, and metabolic pathways. RT–qPCR confirmed increased *RTN4* mRNA expression in COPD and a further increase in patients with COPD and comorbid CAD despite comparable FEV1% predicted between the two COPD groups. **Conclusions:** These findings suggest that elevated *RTN4* expression may serve as a PBMC-derived, monocyte-associated candidate molecular feature related to COPD–CAD comorbidity.

## 1. Introduction

Chronic obstructive pulmonary disease (COPD) is one of the leading causes of morbidity and mortality worldwide and remains a major global public health burden [1,2]. Traditionally defined as a chronic respiratory disorder characterized by persistent airflow limitation, COPD is now increasingly recognized as a heterogeneous systemic disease involving chronic inflammation, immune dysregulation, and multiple extrapulmonary manifestations [3,4]. Beyond progressive pulmonary dysfunction, patients with COPD frequently develop comorbidities that substantially worsen clinical outcomes and long-term prognosis, including cardiovascular disease, lung cancer, and osteoporosis [5,6,7,8]. Importantly, the occurrence of these comorbidities varies among patients with COPD, suggesting potential biological heterogeneity within the COPD population.

Among these comorbidities, cardiovascular disease, particularly coronary artery disease (CAD), is one of the most common and clinically important complications in patients with COPD [9]. Epidemiological studies and meta-analyses have consistently shown that COPD is associated with a significantly increased risk of CAD, myocardial infarction, and cardiovascular mortality, even after adjustment for cardiovascular risk factors such as smoking, age, hypertension, diabetes, and dyslipidemia [9,10,11]. Notably, the relationship between COPD and cardiovascular outcomes appears to follow a dose–response pattern [12]. For every 10% decrease in forced expiratory volume in one second (FEV_1_), the risk of cardiovascular mortality increases by approximately 28%, and the incidence of non-fatal coronary events rises by nearly 20% [13]. These findings suggest that the contribution of COPD to cardiovascular vulnerability cannot be explained solely by shared risk factors, but may instead involve disease-specific systemic mechanisms. Therefore, identifying molecular features that distinguish COPD patients with cardiovascular comorbidity-related biological characteristics may help improve risk stratification and subgroup identification.

Chronic low-grade systemic inflammation is widely considered a central pathophysiological link between COPD and CAD [14,15]. In patients with COPD, persistent peripheral inflammatory activation is accompanied by alterations in circulating immune cell composition and transcriptional programs. These immune abnormalities may contribute to monocyte activation, endothelial dysfunction, vascular inflammation, and atherosclerotic progression [16,17]. However, despite growing recognition of the COPD–CAD association, the molecular features that define COPD patients with a higher likelihood of CAD comorbidity remain poorly understood. In particular, accessible blood-based biomarkers that may help identify COPD subgroups with cardiovascular comorbidity-related inflammatory features are still lacking.

**Figure 1:**
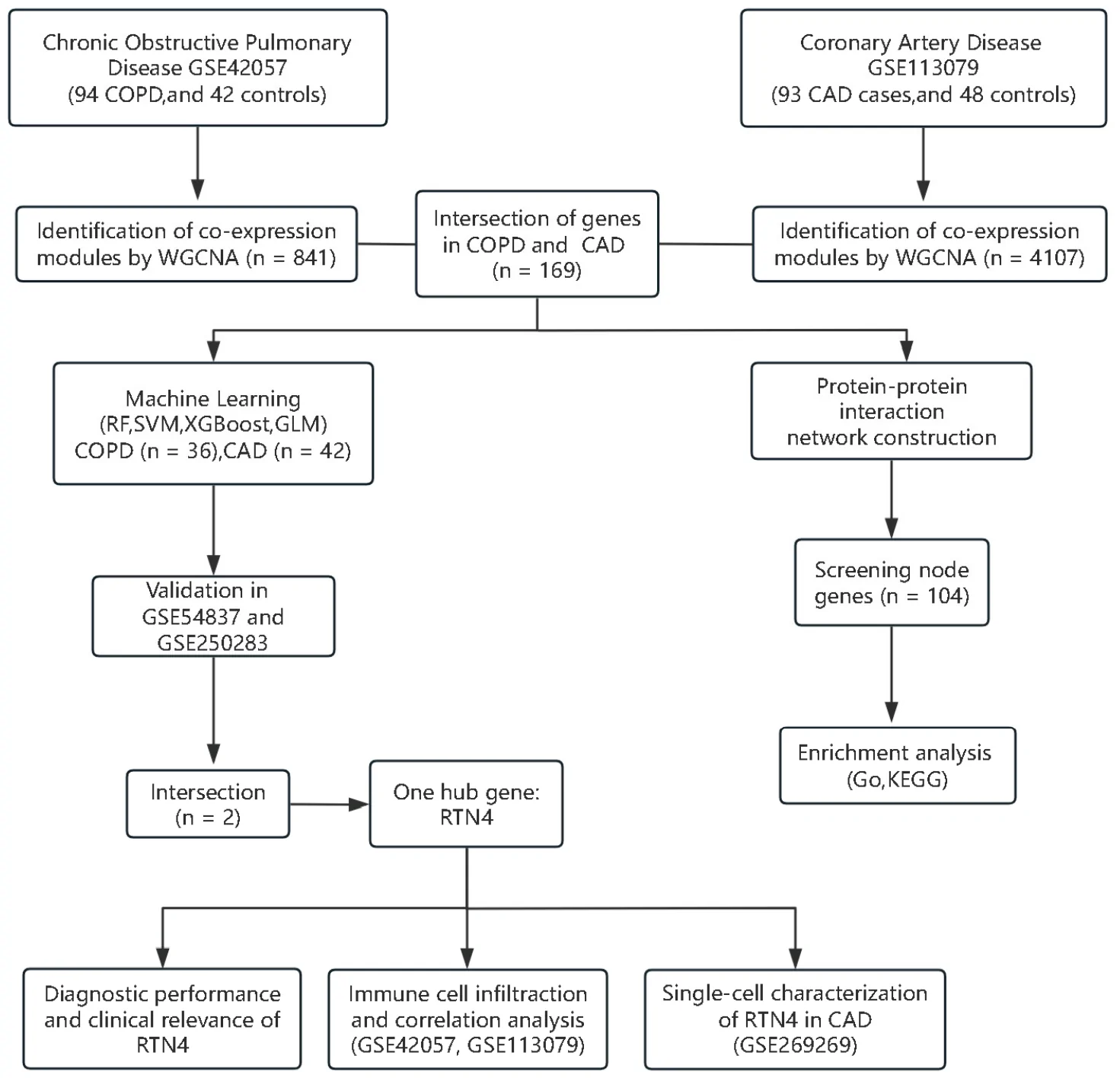
The overall study design.

Peripheral blood mononuclear cells (PBMCs) provide an accessible and informative window into systemic immune and inflammatory states and may capture circulating molecular signals relevant to cardiovascular risk stratification [18,19,20]. Compared with tissue specimens, PBMCs can be obtained more conveniently and safely, making them particularly suitable for investigating systemic inflammatory disorders and developing clinically applicable biomarkers. Previous studies have shown that PBMC-based functional and transcriptomic features can reflect disease-related systemic changes in COPD [21,22,23,24]. In the present study, we analyzed PBMC transcriptomic datasets from independent COPD and CAD cohorts to identify candidate molecular features associated with CAD-related systemic immune alterations in COPD. Candidate genes were further evaluated in relation to estimated immune cell composition and were contextualized at the single-cell level using an independent CAD cohort. The leading candidate gene was subsequently validated in clinical PBMC samples from controls, patients with COPD, and patients with COPD and comorbid CAD. Through this integrative framework, we aimed to identify a candidate PBMC-derived molecular feature associated with COPD–CAD comorbidity. The overall study design is illustrated in Figure 1.

## 2. Materials and Methods

### 2.1. GEO Data Acquisition and Processing

The bulk transcriptomic datasets used in this study were obtained from the Gene Expression Omnibus (GEO) database (https://www.ncbi.nlm.nih.gov/geo/) (accessed on 5 August 2025) using the keywords “COPD” or “CAD”. The inclusion criteria were as follows: (i) human PBMC samples, (ii) high-throughput expression profiling, and (iii) a minimum of 10 samples per group to ensure the reliability of WGCNA. Based on these criteria, four datasets were included: GSE42057, GSE113079, GSE54837, and GSE250283. Among them, GSE42057 (94 COPD patients and 42 healthy controls) [19] and GSE113079 (93 CAD patients and 48 controls) [25] were used as discovery cohorts. GSE54837 (136 COPD patients and 90 controls, with COPD defined as forced expiratory volume in one second to forced vital capacity ratio (FEV1/FVC) < 0.70) [26] and GSE250283 (20 diabetes mellitus [DM] patients with CAD and 21 DM patients without CAD, selected using a covariate-controlled design) [27] were used for independent validation. Detailed information regarding the study population, sample source, platform, disease and control definitions, preprocessing procedures, and batch-correction strategies for each dataset is summarized in Supplementary Table S12. Raw expression data were normalized using the “limma” package in R (v4.4.3) independently according to its corresponding platform and data structure. Probe identifiers were mapped to gene symbols according to the corresponding platform annotation files. The resulting expression matrices, with samples as columns and genes as rows, were used for downstream analyses.

Single-cell RNA sequencing (scRNA-seq) data of PBMCs were retrieved from the GEO database (accession number GSE269269) (accessed on 5 August 2025) [28], which contains samples from patients diagnosed with CAD. The dataset consists of ten acute coronary syndrome (ACS) cases, including five patients with plaque rupture (PR) and five patients without plaque rupture (NPR). This dataset was used to provide CAD-focused cellular context for *RTN4*-associated immune-cell patterns and was not intended as direct single-cell validation of COPD–CAD comorbidity. For subsequent analyses, PR and NPR samples were assigned to the experimental and control groups, respectively. Quality control was performed by retaining cells with 200–6000 detected genes, 500–10,000 unique molecular identifiers (UMIs), and a mitochondrial gene fraction of less than 10%. Filtered gene–barcode matrices were processed and integrated using Seurat (v5.3.0) [29]. Data normalization and scaling were conducted using the NormalizeData() and ScaleData() functions with default parameters. Highly variable genes were identified using FindVariableFeatures(), and the top 3000 variable genes were selected for downstream analyses. Principal component analysis (PCA) was performed, and batch effects across samples were corrected using the Harmony algorithm (harmony v1.2.3) [30]. Unsupervised clustering and dimensionality reduction were carried out based on the Harmony-corrected embeddings using the top 20 dimensions. Cell clusters were visualized using t-distributed stochastic neighbor embedding (t-SNE). Cluster-specific marker genes were identified using the FindAllMarkers() function. Cell types were annotated based on canonical marker genes, including *CD34*, *SPINK2* and *SLAMF7* for CD34^+^ progenitor cells; *PF4* and *PPBP* for megakaryocytes; *CD79A*, *CD79B* and *MS4A1* for B cells; *CD3D*, *NKG7*, *GZMB* and *TRBC1* for T and natural killer (NK) cells; and *CD14*, *LYZ*, *S100A8*, and *S100A9* for monocytes. Monocyte populations were further subsetted for downstream analyses.

### 2.2. Weighted Gene Co-Expression Network Analysis

Weighted gene co-expression network analysis (WGCNA)(v1.73) was performed separately in the GSE42057 and GSE113079 datasets to identify gene modules associated with COPD and CAD. Genes with high expression variability were selected for network construction, and outlier samples were removed by hierarchical clustering. An appropriate soft-thresholding power was chosen based on scale-free topology criteria. Gene co-expression modules were identified using a dynamic tree-cutting approach, with a minimum module size of 30 genes. Module eigengenes were correlated with disease status to identify disease-associated modules. Gene significance (GS) and module membership (MM) were further evaluated, and genes from disease-relevant modules were retained for downstream analyses.

### 2.3. Identification of Overlapping Genes and PPI Network Construction

Genes from disease-associated modules identified by WGCNA in the GSE42057 and GSE113079 datasets were intersected to obtain shared genes, which were visualized using Venn diagrams. The overlapping genes were subsequently uploaded to the Search Tool for the Retrieval of Interacting Genes/Proteins (STRING) database (https://cn.string-db.org/) (accessed on 21 August 2025) to explore potential protein–protein interactions (PPI). The analysis was restricted to Homo sapiens, and interactions with a confidence score greater than 0.4 (medium confidence) were retained to construct the PPI network. The resulting interaction network was exported and visualized using Cytoscape software (version 3.7.2) [31].

### 2.4. Functional Enrichment Analysis

Gene Ontology (GO) and Kyoto Encyclopedia of Genes and Genomes (KEGG) pathway enrichment analyses were performed using the clusterProfiler package in R(v4.4.3), with an adjusted *p* value < 0.05 considered statistically significant.

### 2.5. Machine Learning-Based Identification of Candidate Genes

Multiple machine learning algorithms were used for candidate gene prioritization in the COPD and CAD discovery cohorts. The 169 genes shared between the COPD- and CAD-associated WGCNA modules were used as input features for subsequent machine-learning analyses. Random forest (RF), support vector machine (SVM), extreme gradient boosting (XGBoost) (v1.7.11.1), and generalized linear model (GLM) classifiers were independently constructed in the COPD dataset GSE42057 and the CAD dataset GSE113079, respectively. Model training and evaluation were performed using repeated k-fold cross-validation implemented in the caret package (v7.0.1). Specifically, stratified 5-fold cross-validation was repeated five times to obtain robust estimates of model performance. In each fold, models were trained on four-fifths of the samples and evaluated on the remaining one-fifth. Model performance was evaluated using receiver operating characteristic (ROC) curve analysis(pROC package, v1.19.0.1), with the area under the curve (AUC) calculated for each model. In addition, model interpretability was evaluated using the DALEX package(v2.5.2) to estimate gene importance. Prediction error metrics, including root mean square error (RMSE), were further used to compare the performance of different machine learning models. For each of these three models, the top 20 genes ranked by feature importance were retained, and their union was obtained separately for GSE42057 and GSE113079. The resulting disease-specific candidate gene sets were then intersected to identify genes shared between COPD and CAD.

### 2.6. Validation of Hub Genes and Evaluation of Discriminative Performance

Candidate genes identified by machine learning analysis were further evaluated in independent COPD and CAD datasets by comparing their expression levels between disease and control groups. Genes with significant and directionally consistent differential expression (*p* < 0.05) across the training and validation datasets were considered validated candidate genes. Specifically, GSE54837 and GSE250283 were used as independent validation datasets for COPD and CAD, respectively. The discriminative ability of hub genes was assessed using receiver operating characteristic (ROC) curve analysis(pROC package, v1.19.0.1), and the area under the curve (AUC) was calculated in the corresponding training and validation datasets.

### 2.7. RTN4-Associated Immune Cell Composition Analysis

Immune cell infiltration analysis was performed using the CIBERSORT package (v0.1.0) based on bulk transcriptomic data from the GSE42057 and GSE113079 datasets. Normalized expression matrices were used to estimate the relative proportions of immune cell subsets. Differences between disease and control groups were evaluated using non-parametric statistical tests, and correlations between *RTN4* expression and immune cell proportions were assessed using Spearman correlation analysis, with *p* < 0.05 considered statistically significant.

### 2.8. Metabolic Pathway Activity Assessment

Metabolic pathway activity at the single-cell level was quantified using the scMetabolism package (v0.2.1) in R, with KEGG as the reference metabolic database and AUCell (v1.26.0) as the scoring method. Group-wise pathway activity was summarized using DotPlot.metabolism, and the scaled values returned by the dot plot function were subsequently used for heatmap visualization.

### 2.9. Clinical Sample Collection and PBMC Isolation

Peripheral blood samples were collected from controls, patients with COPD, and patients with COPD complicated with CAD at Shanghai Sixth People’s Hospital Affiliated to Shanghai Jiao Tong University School of Medicine between February 2026 and April 2026. This study was conducted in accordance with the amended Declaration of Helsinki and was approved by the Ethics Committee of Shanghai Sixth People’s Hospital Affiliated to Shanghai Jiao Tong University School of Medicine (Approval No. 2026-YS-127). Written informed consent was obtained from all participants. COPD was diagnosed according to clinical history, respiratory symptoms, and pulmonary function testing, with persistent airflow limitation defined as post-bronchodilator FEV1/FVC < 0.70. CAD was defined based on a documented clinical diagnosis in the medical records, including a previous diagnosis of coronary artery disease, a history of coronary revascularization, or imaging-confirmed CAD defined as ≥50% luminal stenosis in at least one major coronary artery on coronary angiography or coronary computed tomography angiography. Patients with COPD complicated with CAD were assigned to the COPD + CAD group. Control subjects had no history of COPD, CAD, or other major chronic inflammatory diseases and showed no evidence of airflow limitation on pulmonary function testing. Participants with acute infection, autoimmune disease, malignant tumors, or recent systemic corticosteroid or immunosuppressive therapy were excluded.

Peripheral blood samples were collected from enrolled participants into EDTA anticoagulant tubes and processed within 2–4 h after collection. PBMCs were isolated by density-gradient centrifugation using Human Peripheral Blood Lymphocyte Separation Medium (C0025, Beyotime, Shanghai, China) according to the manufacturer’s instructions. Briefly, whole blood was diluted with sterile phosphate-buffered saline (PBS), gently mixed, and carefully layered onto the PBMC separation medium in a sterile centrifuge tube while maintaining a clear interface between the two layers. Samples were centrifuged at 600× *g* for 30 min at room temperature without brake. After centrifugation, the PBMC layer at the plasma–separation medium interface was carefully collected and transferred to a new centrifuge tube. The collected cells were washed twice with PBS at 300× *g* for 10 min. The isolated PBMCs were subsequently used for total RNA extraction.

### 2.10. RNA Extraction and Quantitative Real-Time PCR

Total RNA was isolated from PBMC samples using the EZ-press RNA Purification Kit (EZBioscience, Suzhou, China) according to the manufacturer’s instructions. RNA concentration and purity were evaluated using a NanoDrop One spectrophotometer (Thermo Fisher Scientific, Waltham, MA, USA). Complementary DNA (cDNA) was synthesized from purified RNA using the 4 × EZscript Reverse Transcription Mix II kit (EZBioscience, Suzhou, China). Reverse transcription quantitative PCR (RT–qPCR) was subsequently performed on a QuantStudio 5 Real-Time PCR System (Applied Biosystems, Foster City, CA, USA) using Power SYBR Green Master Mix (Applied Biosystems, Foster City, CA, USA). Each reaction contained cDNA template, forward and reverse primers, SYBR Green Master Mix, and nuclease-free water in a final reaction volume of 10 μL. The relative expression levels of *RTN4* and selected immune activation-related genes were normalized to GAPDH and calculated using the 2^−ΔΔCt^ method. Primer sequences are listed in Supplementary Table S11.

### 2.11. Statistical Analysis

All statistical analyses were performed using R software (v4.4.3). For comparisons between two groups, Student’s *t*-test or the Wilcoxon rank-sum test was applied as appropriate based on data distribution. Comparisons among multiple groups were performed using one-way analysis of variance (ANOVA) or the Kruskal–Wallis test. All tests were two-sided, and a *p* value < 0.05 was considered statistically significant.

## 3. Results

### 3.1. Identification of COPD and CAD Associated Gene Co-Expression Modules by WGCNA

**Figure 2:**
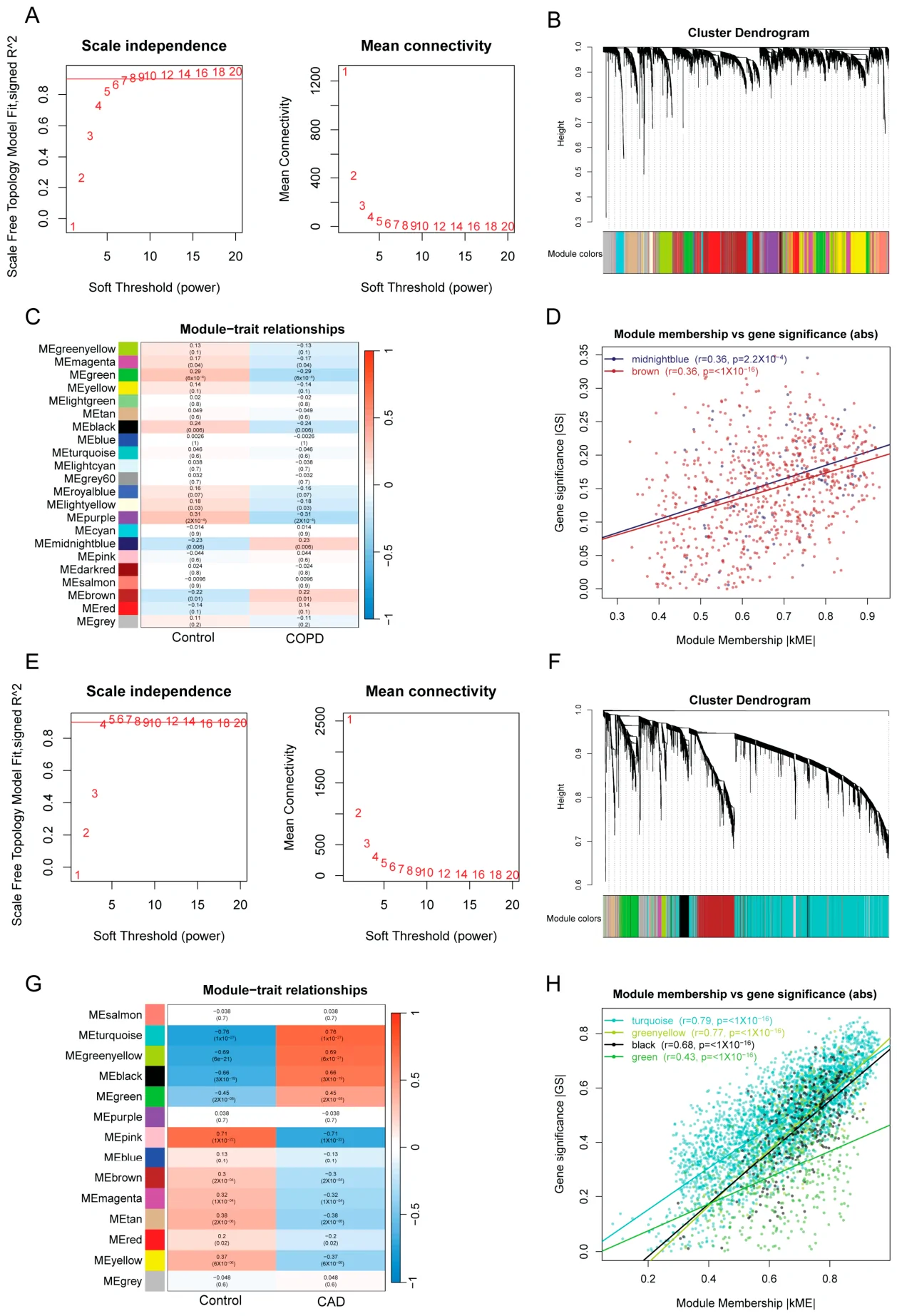
Identification of disease-associated gene modules in COPD and CAD by WGCNA. (**A**) Scale-free topology fit index and mean connectivity across soft-thresholding powers in the COPD dataset (GSE42057). (**B**) Gene dendrogram based on topological overlap in GSE42057, with different colors representing distinct gene co-expression modules identified by WGCNA. (**C**) Heatmap of module–trait relationships in GSE42057. Values in each cell indicate the correlation coefficient and corresponding *p* value. (**D**) Relationships between module membership (MM) and gene significance (GS) for COPD in the midnightblue and brown modules. (**E**) Scale-free topology fit index and mean connectivity across soft-thresholding powers in the CAD dataset (GSE113079). (**F**) Gene dendrogram based on topological overlap in GSE113079, with different colors representing distinct gene co-expression modules identified by WGCNA. (**G**) Heatmap of module–trait relationships in GSE113079. Values in each cell indicate the correlation coefficient and corresponding *p* value. (**H**) Relationships between MM and GS for CAD in the turquoise, greenyellow, black, and green modules.

To identify gene co-expression modules associated with COPD and CAD, WGCNA was performed separately on the GSE42057 and GSE113079 datasets. In GSE42057, soft-threshold analysis indicated that a power of β = 6 best approximated scale-free topology, resulting in 22 co-expression modules (Figure 2A,B). Module–trait analysis identified the midnightblue module (r = 0.23, *p* = 0.006) and the brown module (r = 0.22, *p* = 0.01) as significantly positively correlated with COPD (Figure 2C). These two modules contained a total of 841 genes, including 102 in midnightblue and 739 in brown. MM–GS analysis further showed significant positive correlations in both modules (midnightblue: r = 0.36, *p* = 2.2 × 10^−4^; brown: r = 0.36, *p* < 1 × 10^−16^) (Figure 2D), suggesting that intramodular hub genes were closely associated with COPD. In GSE113079, a soft-thresholding power of β = 4 was selected to construct an approximately scale-free network, yielding 14 co-expression modules (Figure 2E,F). Module–trait analysis showed that the turquoise (r = 0.76, *p* = 1 × 10^−27^), greenyellow (r = 0.69, *p* = 6 × 10^−21^), black (r = 0.66, *p* = 3 × 10^−19^), and green modules (r = 0.45, *p* = 2 × 10^−8^) were significantly positively associated with CAD (Figure 2G). These four modules comprised 4107 genes in total, including 3260 in turquoise, 140 in greenyellow, 306 in black, and 401 in green. MM–GS analysis also revealed significant positive correlations in all four modules (turquoise: r = 0.79; greenyellow: r = 0.77; black: r = 0.68; green: r = 0.43; all *p* < 1 × 10^−16^) (Figure 2H), indicating that hub genes within these modules were strongly related to CAD. Collectively, these disease-associated modules provided the basis for subsequent overlapping gene analysis.

### 3.2. Overlapping Gene Identification and Functional Enrichment Analysis

**Figure 3:**
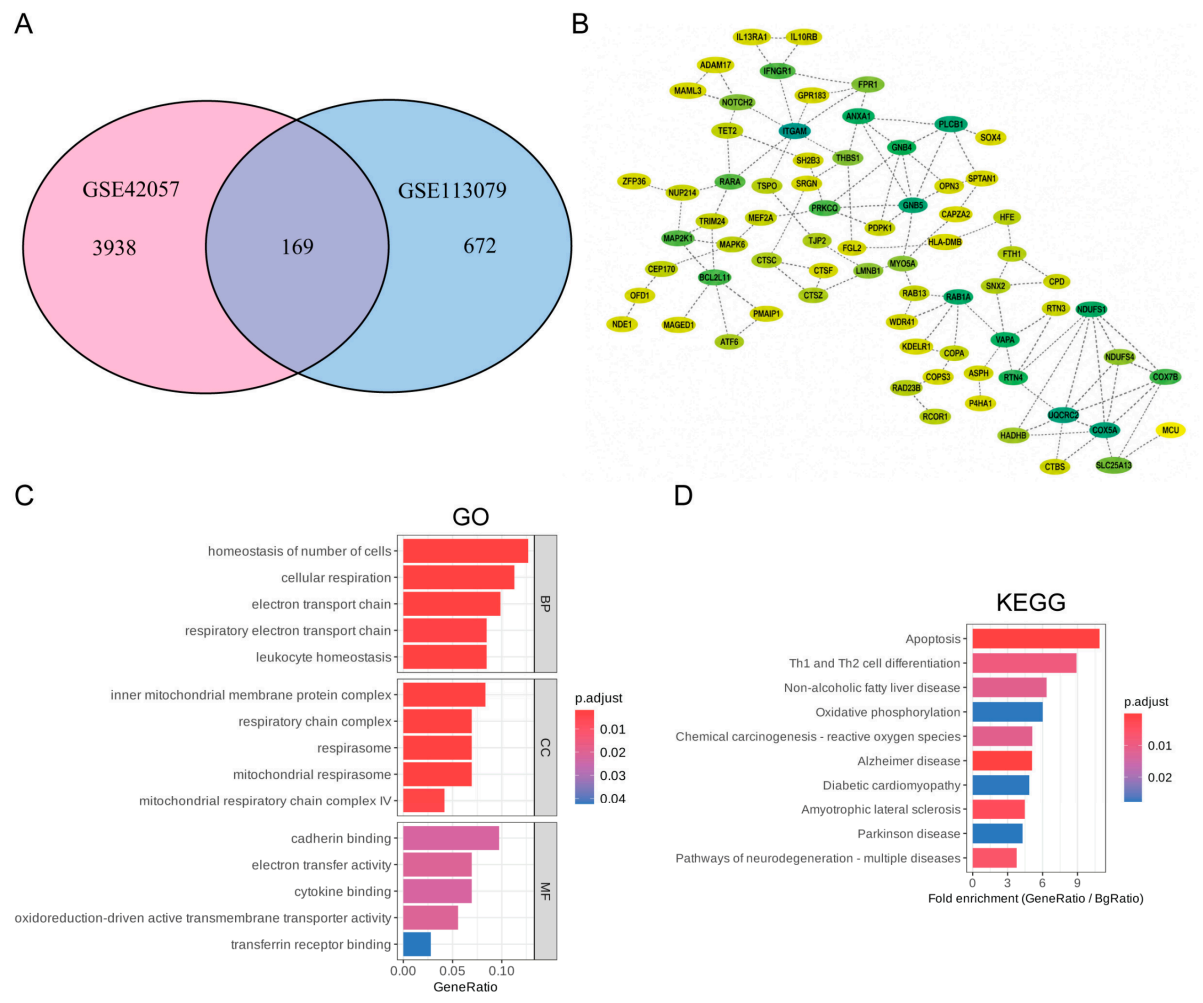
PPI network and functional enrichment of overlapping genes. (**A**) Venn diagram of overlapping genes from COPD (GSE42057, pink) and CAD (GSE113079, blue) modules. (**B**) PPI network of overlapping genes. Node colors indicate the degree of connectivity in the PPI network, with yellow, light green, and dark green representing low, intermediate, and high connectivity, respectively. (**C**,**D**) GO and KEGG enrichment analyses of overlapping genes.

Based on disease-associated modules identified in both datasets, 169 overlapping genes between GSE42057 and GSE113079 were identified by Venn analysis (Figure 3A). The PPI network constructed from these genes retained 104 interacting nodes after removal of non-interacting proteins (Figure 3B). Functional enrichment analysis was performed on 104 PPI genes to explore potential shared molecular mechanisms between COPD and CAD. GO analysis revealed significant enrichment in biological processes related to cellular population homeostasis, cellular respiration, the electron transport chain, and leukocyte homeostasis. At the cellular component level, these genes were primarily enriched in mitochondrial inner membrane protein complex, respiratory chain complex, and mitochondrial respirasome. Molecular function analysis indicated enrichment in cadherin binding, electron transfer activity, and cytokine binding (Figure 3C). KEGG pathway analysis further demonstrated significant enrichment in apoptosis, Th1 and Th2 cell differentiation, oxidative phosphorylation, and multiple disease pathways associated with oxidative stress and energy metabolism (Figure 3D). Collectively, these results suggest that mitochondrial dysfunction, disrupted energy metabolism, and aberrant immune regulation may represent shared molecular features underlying the comorbidity between COPD and CAD.

### 3.3. Machine-Learning Prioritization and Validation of Candidate Genes

**Figure 4:**
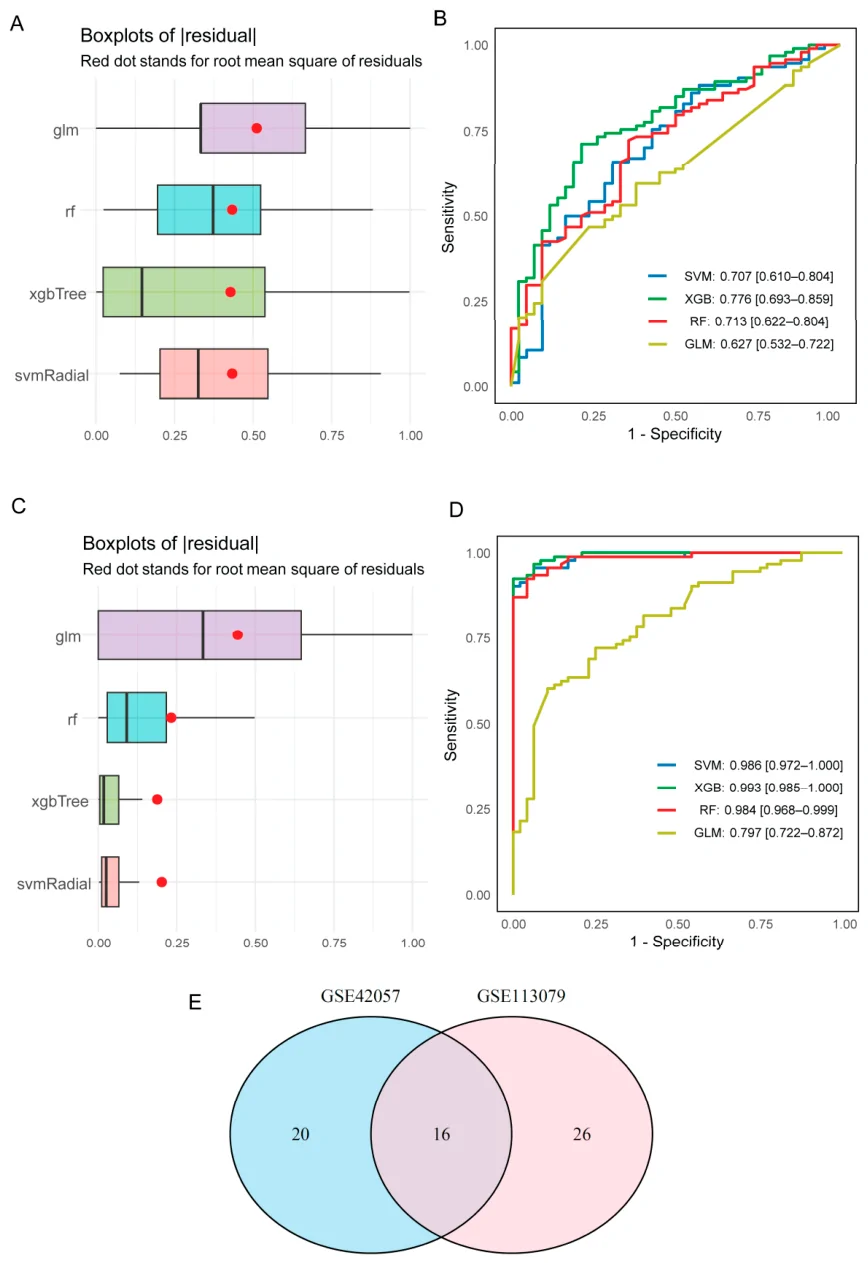
Machine-learning model performance and feature prioritization in COPD and CAD discovery cohorts. (**A**,**C**) Residual distributions of generalized linear model (GLM), random forest (RF), extreme gradient boosting (XGBoost), and support vector machine (SVM) models in the COPD (GSE42057) and CAD (GSE113079) discovery cohorts, respectively; red dots indicate root mean square error (RMSE) values. (**B**,**D**) Receiver operating characteristic (ROC) curves of the four models in GSE42057 and GSE113079, respectively, with area under the curve (AUC) values and 95% confidence intervals (CIs) shown in the legends. (**E**) Intersection of candidate features independently prioritized in the two discovery cohorts.

**Figure 5:**
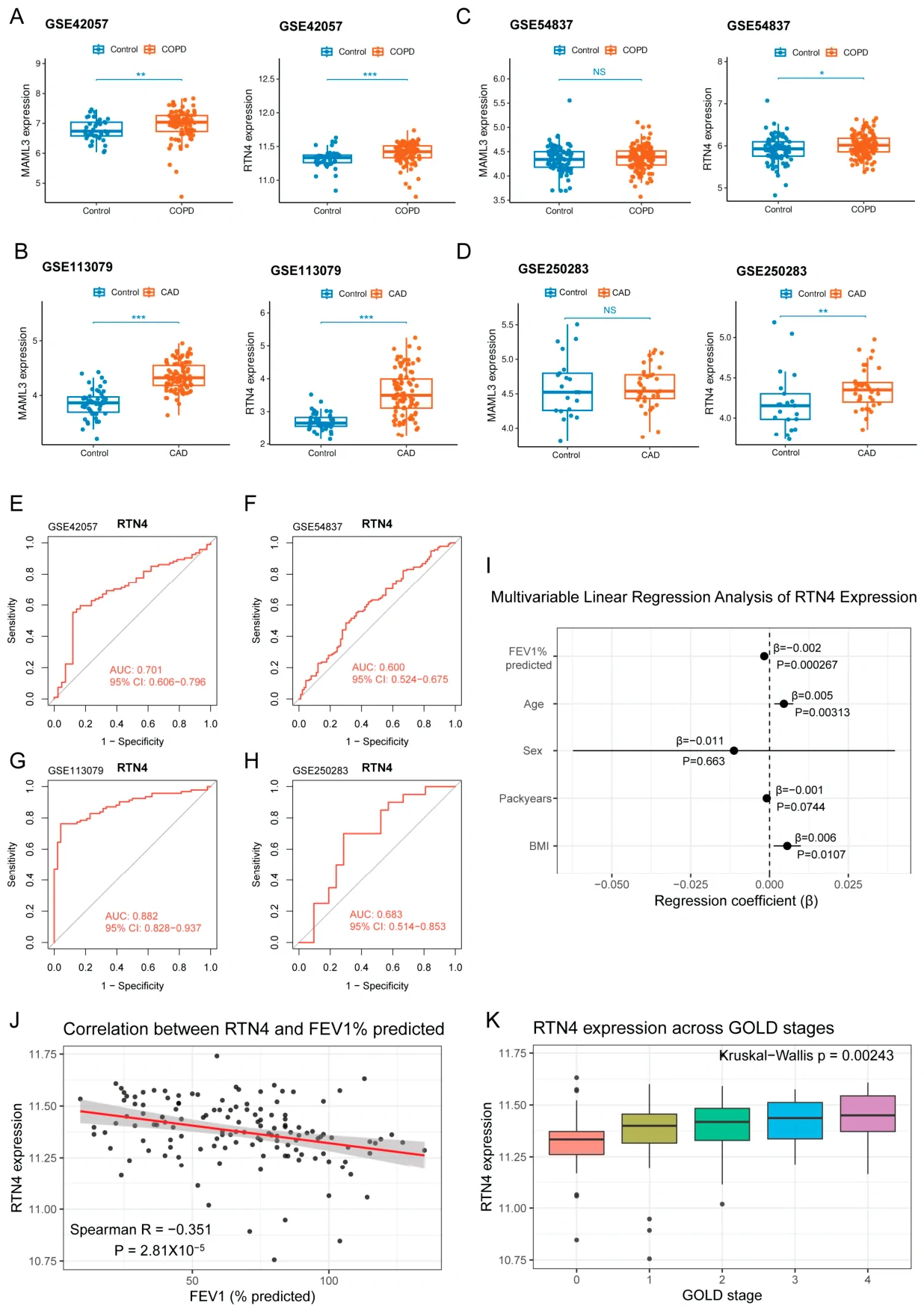
Expression patterns, discriminative performance, and clinical relevance of *RTN4* in COPD and CAD. (**A**,**B**) Expression levels of *MAML3* and *RTN4* in the COPD training dataset GSE42057 and CAD training dataset GSE113079, respectively. (**C**,**D**) Validation of *MAML3* and *RTN4* expression in the independent COPD dataset GSE54837 and CAD dataset GSE250283, respectively. Each point represents an individual sample. (**E**–**H**) ROC curve analyses evaluating the discriminative performance of *RTN4* in GSE42057 (**E**), GSE54837 (**F**), GSE113079 (**G**), and GSE250283 (**H**); AUC values and 95% confidence intervals are indicated. (**I**) Multivariable linear regression analysis assessing the association between *RTN4* expression and FEV1% predicted, age, sex, smoking pack-years, and body mass index (BMI). (**J**) Spearman correlation analysis between *RTN4* expression and FEV1% predicted. The red solid line represents the fitted regression line, and the grey shaded area indicates the 95% confidence interval. (**K**) Comparison of *RTN4* expression levels across different GOLD stages of COPD using the Kruskal–Wallis test. * *p* < 0.05, ** *p* < 0.01, *** *p* < 0.001; NS, not significant.

To identify candidate genes associated with COPD and CAD, four machine learning models were constructed and evaluated in GSE42057 and GSE113079 using residual distributions and ROC curve analysis. As shown in Figure 4A–D, the SVM, XGBoost, and RF models exhibited comparable model performance. Based on the feature importance rankings derived from RMSE, the top 20 genes predicted by RF, SVM, and XGBoost were selected from each dataset. The union of the selected genes yielded 36 candidate genes in GSE42057 and 42 in GSE113079. Intersection analysis of these two gene sets identified 16 genes shared between COPD and CAD (Figure 4E). After evaluating differential expression levels and expression trends across the training datasets, two candidate genes, mastermind-like transcriptional coactivator 3 (*MAML3*) and *RTN4*, were retained (Figure 5A,B). Differential expression of both genes was confirmed in external validation cohorts of COPD (GSE54837) and CAD (GSE250283). Notably, *RTN4* showed consistent differential expression changes across the corresponding training and validation datasets (*p* < 0.05), and was therefore selected as the primary candidate gene for subsequent analyses (Figure 5C,D). To further evaluate the discriminative performance of *RTN4*, ROC analysis was performed in both the training and external validation datasets. *RTN4* showed modest to moderate discriminative performance in COPD cohorts, with AUC values of 0.701 in GSE42057 and 0.600 in GSE54837, and showed higher discriminative performance in CAD cohorts, particularly in GSE113079 (AUC = 0.882) and GSE250283 (AUC = 0.683) (Figure 5E–H). The variability in AUC values across cohorts suggests that the discriminative performance of *RTN4* may be influenced by disease stage, cohort heterogeneity, and clinical characteristics. To further assess the clinical relevance of *RTN4*, we examined its association with pulmonary function in the GSE42057 cohort. Multivariable linear regression analysis showed that *RTN4* expression remained significantly negatively associated with FEV1% predicted (β = −0.002, *p* = 0.000267) after adjustment for age, sex, pack-years, and BMI. In addition, age (β = 0.005, *p* = 0.00313) and BMI (β = 0.006, *p* = 0.0107) were positively associated with *RTN4* expression, whereas sex was not significantly associated (*p* = 0.663) and pack-years showed a borderline association (*p* = 0.0744) (Figure 5I). Consistently, Spearman correlation analysis also demonstrated a significant negative correlation between *RTN4* expression and FEV1% predicted (R = −0.351, *p* = 2.81 × 10^−5^) (Figure 5J). Moreover, *RTN4* expression increased progressively with advancing GOLD stages, with significant differences observed across disease stages (*p* = 0.00243) (Figure 5K).

### 3.4. Association Between Estimated Peripheral Immune Cell Composition and RTN4 Expression

**Figure 6:**
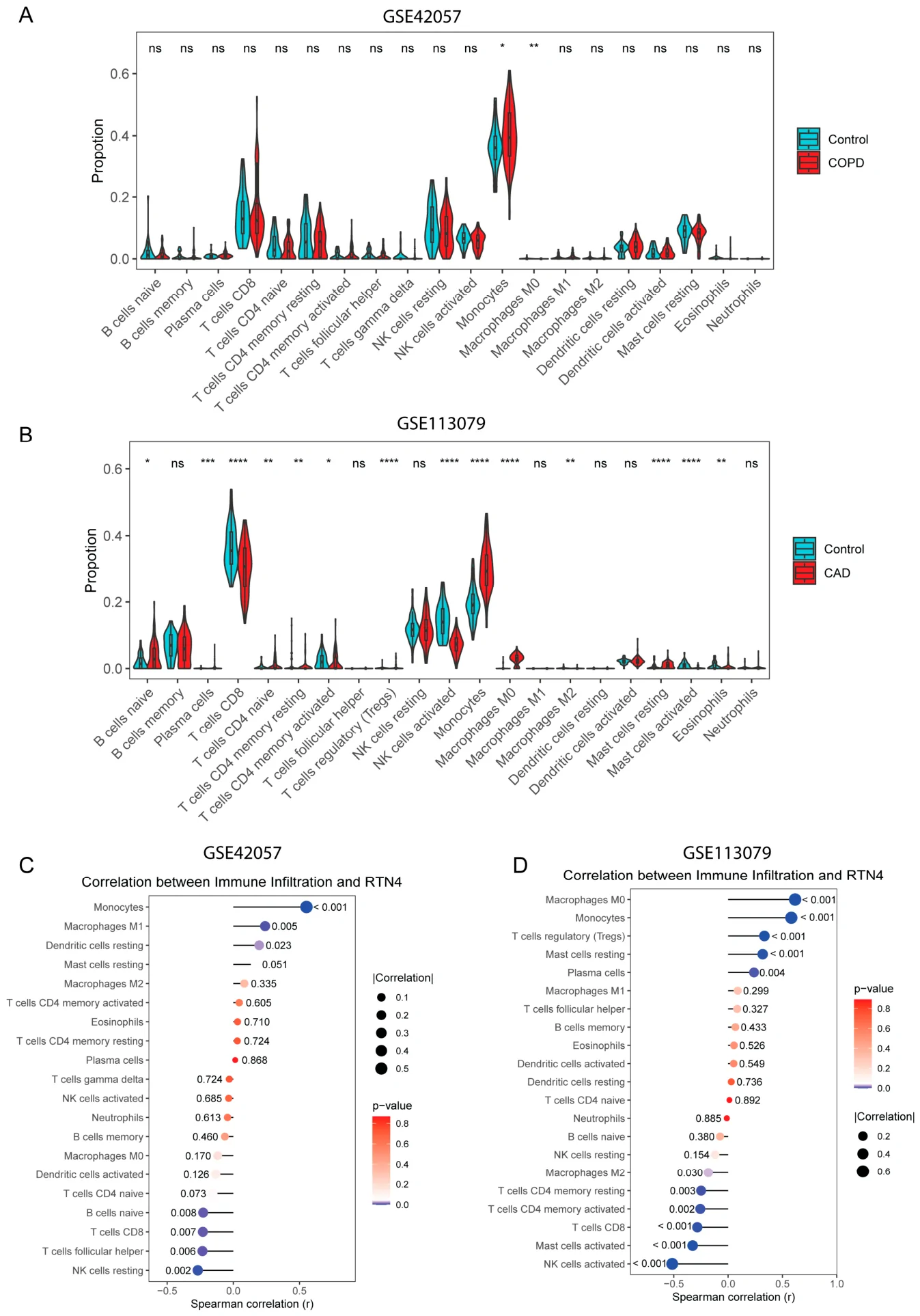
Immune landscape and RTN4-associated infiltration patterns in COPD and CAD. (**A**,**B**) Violin plots showing the estimated proportions of immune cell subsets between control and disease groups in the COPD cohort GSE42057 and CAD cohort GSE113079, respectively. Differences between groups were assessed using the Wilcoxon rank-sum test. (**C**,**D**) Spearman correlation analyses between RTN4 expression and estimated immune cell proportions in GSE42057 and GSE113079, respectively. The direction and length of the horizontal lines indicate the direction and magnitude of the Spearman correlation coefficient, respectively; dot size represents the absolute correlation coefficient, and dot color indicates the corresponding *p* value. Values of *p* < 0.001 are shown as “<0.001”. ns, not significant (*p* ≥ 0.05); * *p* < 0.05; ** *p* < 0.01; *** *p* < 0.001; **** *p* < 0.0001.

To characterize estimated peripheral immune cell composition in COPD, the CIBERSORT algorithm was applied to the GSE42057 dataset to estimate the relative proportions of 22 immune cell subsets. The overall immune cell distribution across individual samples is shown in Supplementary Figure S1A. Most immune cell populations did not differ significantly between COPD patients and controls; however, the estimated proportions of monocytes and M0 macrophages were significantly increased in the COPD group (*p* < 0.05), indicating an altered peripheral myeloid cell composition in COPD (Figure 6A). To assess estimated immune cell composition patterns in CAD, CIBERSORT analysis was similarly performed in the GSE113079 dataset to characterize estimated immune cell composition in CAD, with the overall distribution shown in Supplementary Figure S1B. CAD patients exhibited significant changes in multiple immune cell subsets, including increased proportions of monocytes and M0 macrophages, as well as alterations in several T cell and NK cell subsets, suggesting broad remodeling of peripheral immune composition (Figure 6B). To further explore the relationship between *RTN4* expression and estimated immune cell composition, Spearman correlation analysis was conducted between *RTN4* expression levels and the estimated proportions of immune cell subsets. In the GSE42057 dataset, *RTN4* expression showed positive correlations with monocytes and selected myeloid cell subsets, while associations with lymphoid populations were relatively weak (Figure 6C). In the GSE113079 dataset, *RTN4* expression was likewise positively correlated with monocytes and M0 macrophages and negatively correlated with several T cell and NK cell subsets (Figure 6D). Notably, although the overall estimated immune cell composition patterns differed between COPD and CAD, *RTN4* expression showed a consistent positive correlation with monocyte proportions in both datasets, suggesting an association between *RTN4* expression and peripheral monocyte-related immune features.

**Figure 7:**
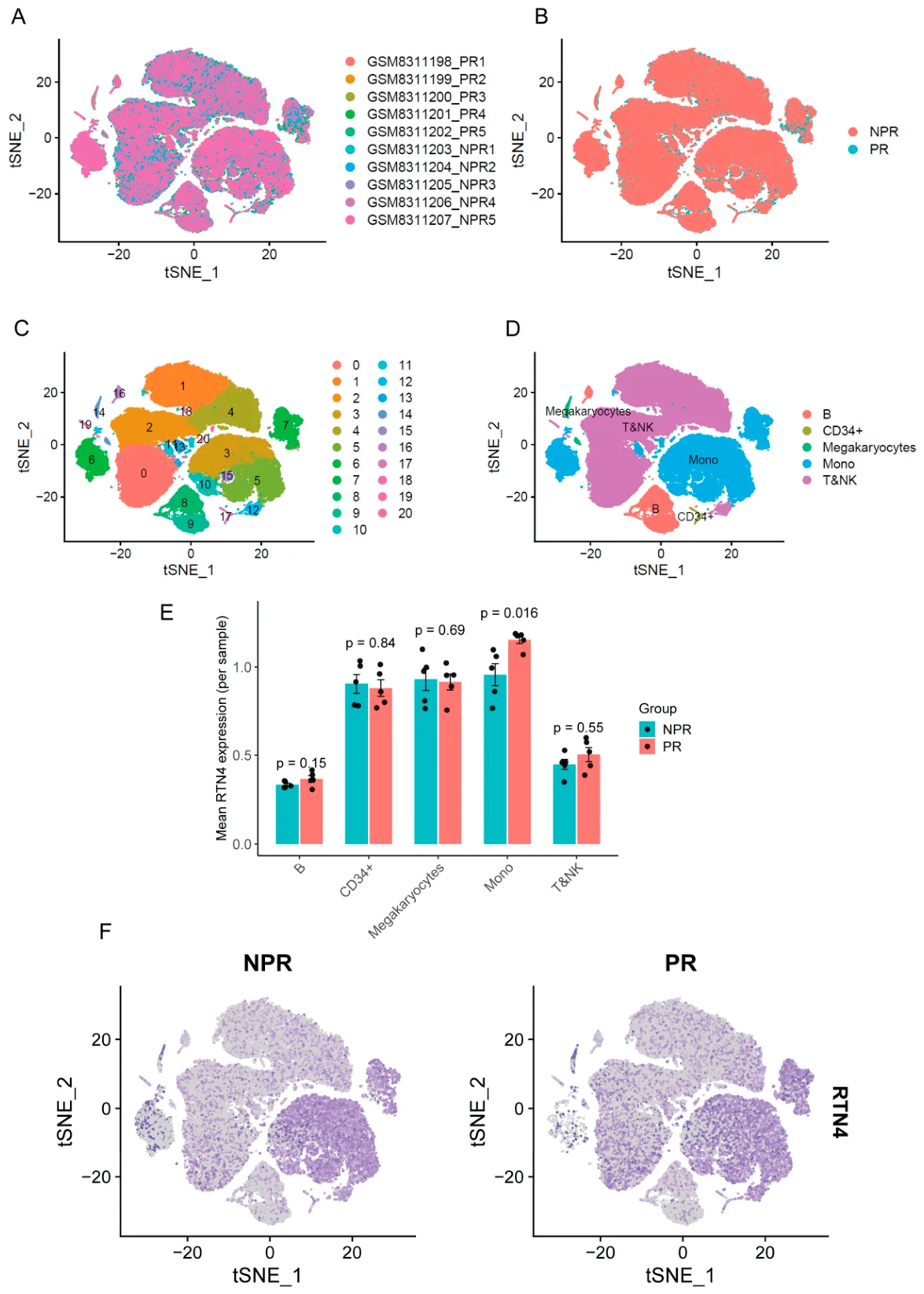
Single-cell expression patterns and cell type–specific distribution of *RTN4* in PR and NPR samples. (**A**) t-SNE plot colored by sample origin, showing good mixing of cells across samples in low-dimensional space. (**B**) t-SNE plot colored by clinical group: NPR, patients without plaque rupture; PR, patients with plaque rupture. (**C**) t-SNE plot showing unsupervised clustering, identifying 21 cell clusters. (**D**) t-SNE plot after cell type annotation, revealing five major immune cell populations: B cells, CD34^+^ cells, megakaryocytes, monocytes, and T&NK cells. (**E**) Comparison of mean *RTN4* expression between NPR and PR groups across different cell types, aggregated at the sample level; data are presented as mean ± SEM, and *p* values are calculated using the Wilcoxon rank-sum test. (**F**) Feature plots of *RTN4* expression in NPR and PR samples.

**Figure 8:**
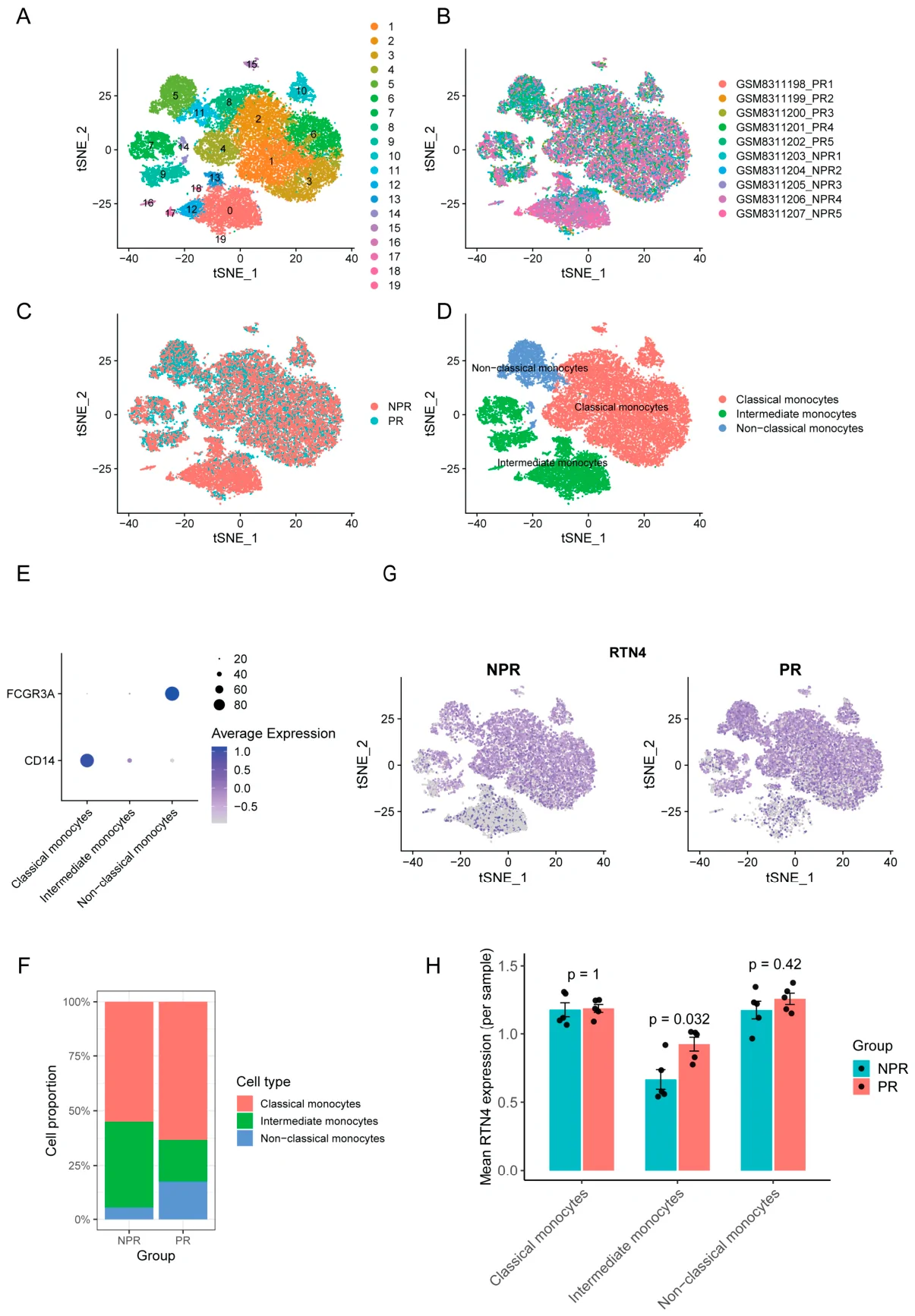
Monocyte subset delineation and *RTN4* expression in PR and NPR samples. (**A**) t-SNE plot showing unsupervised clustering of monocytes. (**B**) t-SNE plot colored by sample origin. (**C**) t-SNE plot colored by clinical group (NPR vs. PR). (**D**) t-SNE plot of monocyte subsets annotated based on canonical markers, including classical, intermediate, and non-classical monocytes. (**E**) Dot plot of CD14 and FCGR3A expression across monocyte subsets. (**F**) Proportional composition of monocyte subsets in NPR and PR groups. (**G**) Feature plots showing *RTN4* expression in NPR and PR samples. (**H**) Comparison of *RTN4* expression between NPR and PR groups across monocyte subsets after sample-level aggregation; values are presented as means, and *p* values are calculated using the Wilcoxon rank-sum test.

To characterize the cellular landscape associated with plaque instability in CAD, PBMC single-cell transcriptomic data from PR and NPR samples were integrated for analysis. Unsupervised clustering identified 21 clusters, which were further annotated into five major cell types: B cells, CD34^+^ cells, megakaryocytes, monocytes, and T&NK cells. PR and NPR cells largely overlapped in low-dimensional space, indicating no major global differences in cellular distribution between the two groups (Figure 7A–D). Sample-level analysis of *RTN4* expression across major cell types showed no significant differences between PR and NPR samples in B cells, CD34^+^ cells, megakaryocytes, or T&NK cells. However, *RTN4* expression was significantly higher in monocytes from PR samples than in those from NPR samples (*p* = 0.016) (Figure 7E). Consistently, feature plots showed that *RTN4* expression was mainly enriched in monocyte regions and was overall higher in PR samples (Figure 7F). Given that *RTN4* upregulation was predominantly localized to monocytes, we next performed refined clustering of the monocyte compartment. After clustering, monocytes were visualized by t-SNE, showing distinct clusters and broad distribution across individual samples and PR/NPR groups (Figure 8A–C). Based on canonical marker expression, monocytes were classified into classical, intermediate, and non-classical subsets (Figure 8D,E).

### 3.5. RTN4-Associated Signals Are Enriched in Monocyte Subsets in CAD Single-Cell Data

The distribution of monocyte subsets was then compared between PR and NPR groups, showing some variation in subset proportions between the two groups (Figure 8F). Feature plot visualization showed detectable *RTN4* expression within the monocyte compartment in both NPR and PR samples (Figure 8G). Further comparison of *RTN4* expression within each monocyte subset showed no significant differences between PR and NPR samples in classical monocytes or non-classical monocytes. In contrast, *RTN4* expression was significantly increased in intermediate monocytes from PR samples compared with NPR samples (*p* = 0.032) (Figure 8H). Notably, intermediate monocytes were not more abundant in PR samples, indicating that the increased *RTN4* signal in this subset is unlikely to be driven by expansion of the intermediate monocyte population. Instead, it more likely reflects higher *RTN4* expression within intermediate monocytes themselves. These findings suggest that the PR-associated increase in *RTN4* expression is most evident in intermediate monocytes, representing a subset-related expression change rather than a broad or composition-driven alteration across all monocyte subsets.

### 3.6. RTN4-High Intermediate Monocytes Are Associated with Immune Activation- and Metabolic Remodeling-Related Transcriptional Programs in CAD

**Figure 9:**
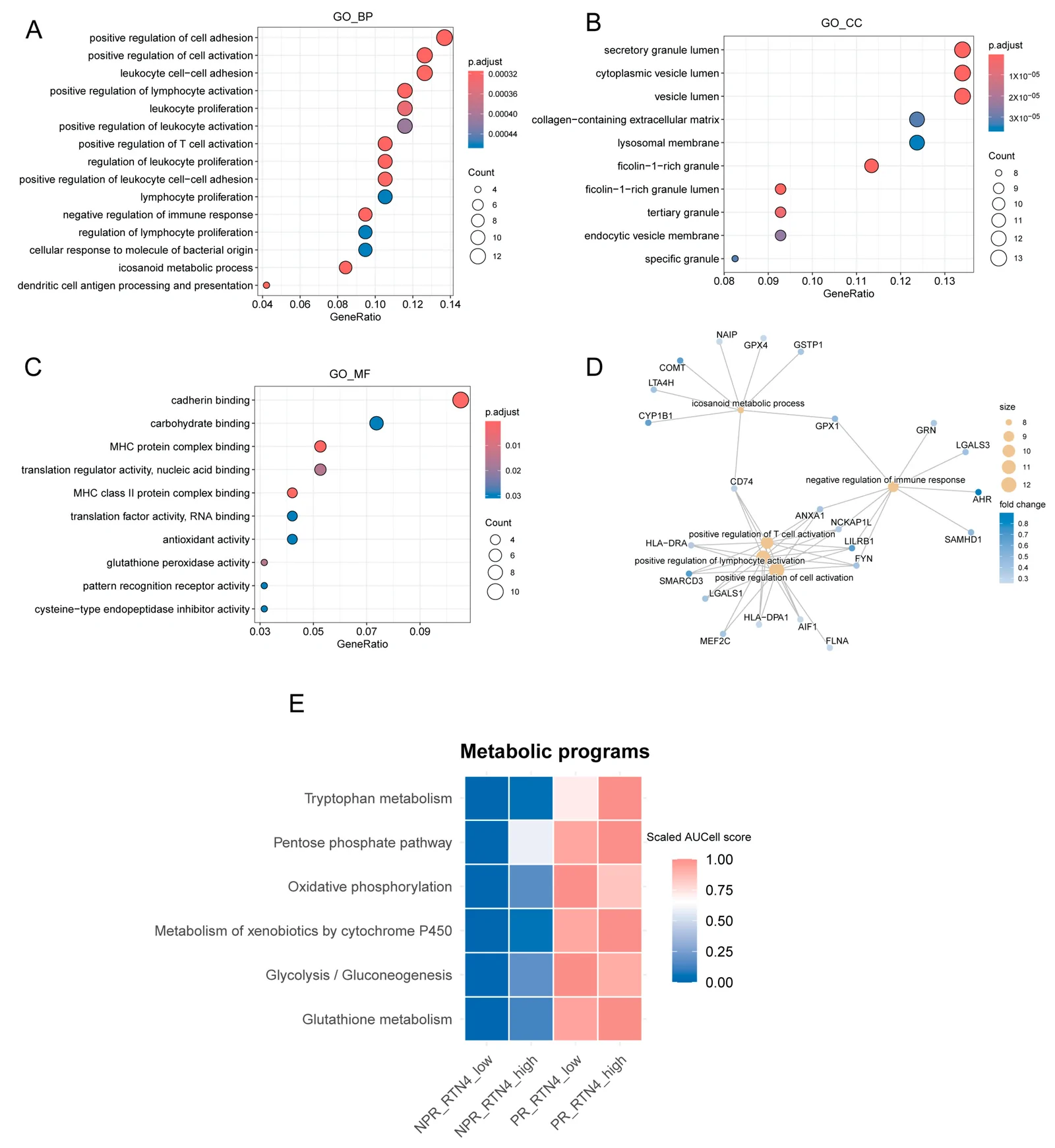
*RTN4*-associated functional enrichment and metabolic programs. (**A**–**C**) GO enrichment analyses of differentially expressed genes between *RTN4*-high and *RTN4*-low intermediate monocytes. Bubble size indicates the number of enriched genes, and color represents adjusted *p* values. (**D**) Gene–pathway network constructed from GO biological process terms. (**E**) Heatmap showing scaled group-wise AUCell scores of representative metabolic programs across the NPR_*RTN4*_low, NPR_*RTN4*_high, PR_*RTN4*_low, and PR_*RTN4*_high groups. Values were derived from DotPlot.metabolism.

To characterize functional alterations associated with *RTN4* upregulation in intermediate monocytes, differential gene expression analysis was performed between *RTN4*-high and *RTN4*-low cells, followed by functional enrichment analyses. GO biological process analysis revealed that genes upregulated in *RTN4*-high intermediate monocytes were predominantly enriched in immune activation–related processes, including leukocyte activation, cell activation, immune cell adhesion, and lymphocyte proliferation (Figure 9A). GO cellular component analysis showed enrichment of these genes in vesicle- and lysosome-associated compartments, such as secretory granule lumen, cytoplasmic vesicle lumen, and lysosomal membrane (Figure 9B), while molecular function analysis revealed significant enrichment in antigen presentation–related binding functions, cadherin binding, translation-regulatory activity, and antioxidant activity (Figure 9C). Gene–pathway network analysis revealed that immune-related pathways, particularly those involved in antigen processing and presentation, occupied central positions within the regulatory network and were closely connected with key immune regulatory genes, including *CD74*, *HLA-DRA*, *HLA-DPA1*, *LGALS1*, and *FYN* (Figure 9D). In parallel, metabolic activity scoring showed coordinated upregulation of multiple metabolic programs in *RTN4*-high intermediate monocytes, particularly in PR samples. Notably, higher activity scores were observed for tryptophan metabolism, the pentose phosphate pathway, xenobiotic metabolism via cytochrome P450, and glutathione metabolism, collectively revealing transcriptional patterns consistent with altered redox-related metabolism and immunometabolic states (Figure 9E). Collectively, these results indicate that *RTN4*-high intermediate monocytes are associated with immune activation and metabolism related transcriptional programs under PR conditions.

### 3.7. Clinical PBMC RT–qPCR Validation of RTN4 Expression in COPD Patients With or Without CAD

**Figure 10:**
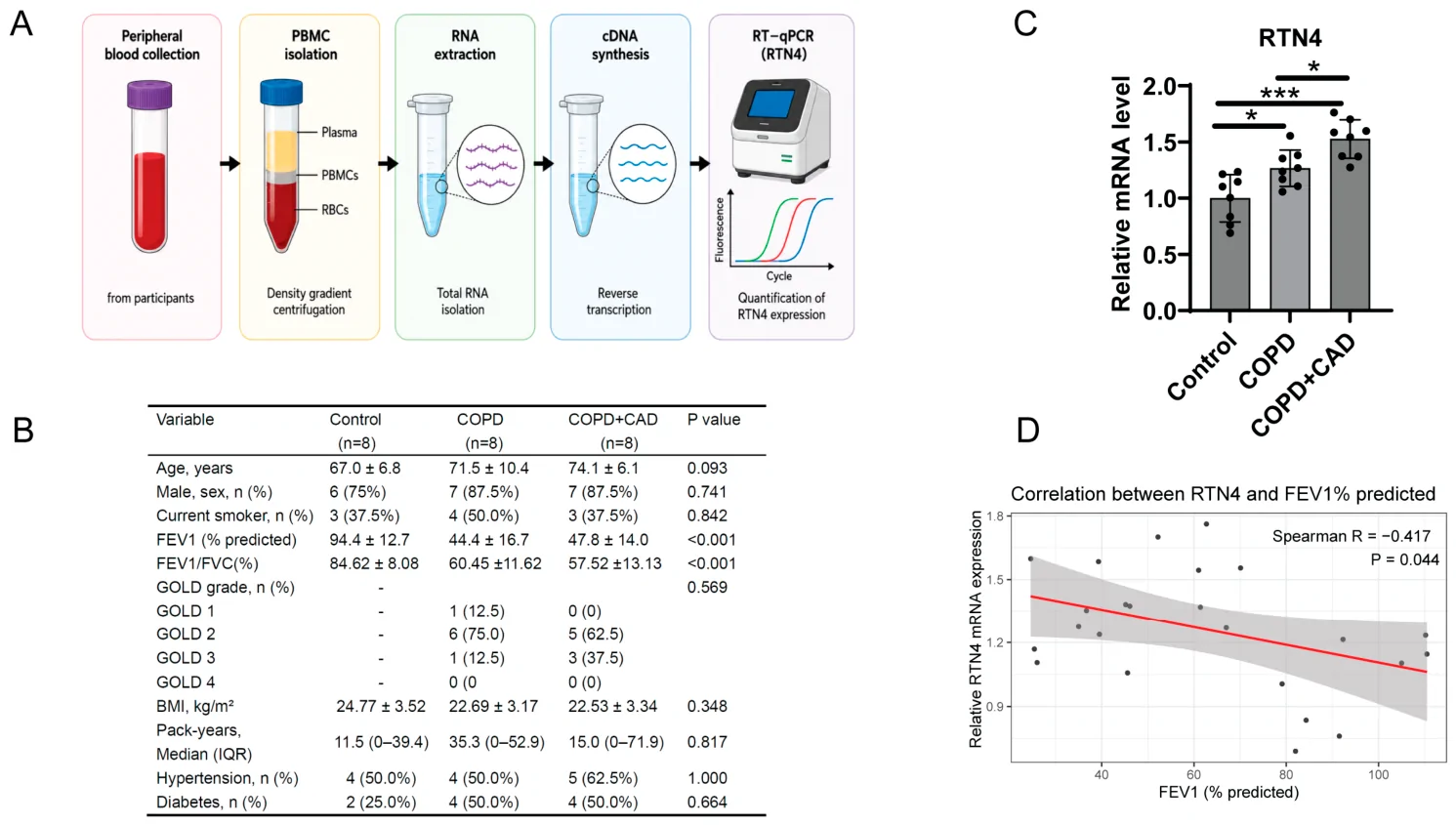
Clinical validation of *RTN4* expression in PBMCs from controls, COPD patients, and COPD patients with CAD. (**A**) Schematic overview of the clinical validation workflow. (**B**) Clinical characteristics of the validation cohort, including controls without a clinical diagnosis of COPD or CAD, patients with COPD, and patients with COPD and comorbid CAD. Data are presented as mean ± standard deviation (SD), median (interquartile range [IQR]), or n (%), as appropriate. Continuous variables were compared using one-way analysis of variance (ANOVA) or the Kruskal–Wallis test, as appropriate. Categorical variables were compared using Fisher’s exact test. GOLD grade was compared between the COPD and COPD+CAD groups. A two-sided *p* value < 0.05 was considered statistically significant. (**C**) Relative *RTN4* mRNA expression in PBMCs from the three groups. Statistical significance was assessed using one-way ANOVA followed by Tukey’s multiple comparisons test. (**D**) Spearman correlation between *RTN4* expression and FEV1% predicted. The red line represents a fitted linear trend for visualization, and the grey shaded area indicates the corresponding 95% confidence interval. Spearman correlation coefficients and *p* values are shown in the plot. * *p* < 0.05, *** *p* < 0.001.

To assess the clinical relevance of *RTN4*, we performed RT–qPCR validation in an institutional PBMC cohort comprising 8 controls without a clinical diagnosis of COPD or CAD, 8 patients with COPD, and 8 patients with COPD and comorbid CAD. PBMCs were isolated from peripheral blood samples, and *RTN4* mRNA expression was quantified by RT–qPCR. The clinical validation workflow is shown in Figure 10A. The baseline characteristics of this cohort are summarized in Figure 10B. There were no significant differences among the three groups in age, sex distribution, current smoking status, BMI, smoking exposure expressed as pack-years, hypertension, or diabetes. As expected, patients in both COPD groups showed markedly reduced FEV1% predicted and FEV1/FVC compared with controls. Notably, FEV1% predicted was comparable between patients with COPD and those with COPD and comorbid CAD. Similarly, the distribution of GOLD grades did not differ significantly between the COPD and COPD+CAD groups (*p* = 0.569). These findings suggest that These findings suggest that the COPD+CAD group was not characterized by substantially greater airflow limitation than the COPD group alone. RT–qPCR analysis showed that *RTN4* mRNA expression was increased in PBMCs from patients with COPD compared with controls and was further elevated in patients with COPD and comorbid CAD (Figure 10C). Moreover, Spearman correlation analysis demonstrated a significant negative correlation between *RTN4* expression and FEV1% predicted across all participants (R = −0.417, *p* = 0.044; Figure 10D). Collectively, these findings provide preliminary clinical evidence that *RTN4* is upregulated in PBMCs from patients with COPD, particularly in those with comorbid CAD, and is associated with reduced FEV1% predicted. Notably, *RTN4* expression was further increased in patients with COPD and comorbid CAD despite comparable lung function between the two COPD groups, suggesting that the higher *RTN4* expression in the COPD+CAD group was unlikely to be explained solely by differences in airflow limitation.

## 4. Discussion

Epidemiological studies have consistently demonstrated that COPD is independently linked to a higher incidence of cardiovascular disease, with an increased risk of CAD and cardiovascular mortality, even after adjustment for smoking and other confounders [32,33,34]. COPD is increasingly recognized as a chronic pulmonary disease accompanied by systemic inflammation, whereas CAD is a chronic inflammatory vascular disease rooted in atherosclerosis. PBMCs, which comprise monocytes, lymphocytes, and other mononuclear immune cells, provide a biologically relevant window into systemic inflammatory alterations [35,36,37]. In this context, our integrative analysis of PBMC transcriptomic cohorts identified overlapping COPD- and CAD-associated molecular networks. These shared genes were mainly enriched in pathways related to mitochondrial function, energy metabolism, immune regulation, and metabolic remodeling, suggesting that COPD and CAD may share systemic immunometabolic alterations at the peripheral blood level. Through combined PPI analysis and machine-learning–based feature prioritization, *RTN4* was consistently identified within this shared network and showed stable upregulation across independent cohorts. These findings suggest that *RTN4* may represent a shared peripheral blood molecular feature of COPD and CAD rather than a disease-specific diagnostic marker. In COPD datasets, higher *RTN4* expression was associated with impaired pulmonary function, as reflected by its negative correlation with FEV1% predicted and its progressive increase across higher GOLD stages.

*RTN4*, also known as Nogo, is a member of the reticulon protein family and is primarily localized to the endoplasmic reticulum (ER) membrane, where it contributes to ER structural integrity, membrane curvature, and cellular stress responses [38]. Through mitochondria-associated membranes (MAMs), the ER is closely linked to metabolic regulation and oxidative stress responses [39,40]. *RTN4* comprises several major isoforms, including Nogo-A (*RTN4A*), Nogo-B (*RTN4B*), and Nogo-C (*RTN4*C), which share a common C-terminal region but differ in their N-terminal domains [41]. Although *RTN4* was initially characterized in the context of neural development [42,43,44], accumulating evidence supports its involvement in immune regulation. In bone marrow-derived macrophages, Nogo-A participates in LPS-induced inflammatory activation, whereas Nogo deficiency reduces C/EBP homologous protein (CHOP) and pro-inflammatory mediator expression and impairs macrophage migration and phagocytosis [45]. Similarly, Nogo-B has been implicated in macrophage inflammatory responses through TLR4-associated MAPK signaling and regulation of inflammatory cytokine production and cell migration [46]. In addition, Nogo-B is highly expressed in vascular endothelial cells and vascular smooth muscle cells and contributes to vascular homeostasis and remodeling [47,48]. Endothelial Nogo-B has been shown to facilitate ICAM-1-dependent transmigration of monocytes and neutrophils, thereby linking vascular *RTN4* signaling to leukocyte recruitment and acute inflammation [48]. More recent studies further suggest that endothelial Nogo-B can influence ER–mitochondrial communication, mitochondrial ROS generation, adhesion molecule expression, and inflammatory signaling during coronary atherosclerosis [49]. Nogo-C, the shortest classical isoform, has also been implicated in cardiovascular injury, with experimental studies showing that its deficiency attenuates cardiomyocyte apoptosis and improves cardiac function after myocardial infarction [50]. Together, these findings indicate that *RTN4* biology extends beyond neural growth inhibition and encompasses macrophage inflammatory activation, leukocyte trafficking, endothelial dysfunction, and cardiovascular stress responses. These observations are consistent with our finding that *RTN4*-related alterations were particularly evident in the monocyte compartment and support the possibility that elevated *RTN4* reflects a monocyte-associated inflammatory state relevant to COPD–CAD comorbidity. Nevertheless, the bulk transcriptomic and RT–qPCR analyses used in the present study assessed *RTN4* at the gene level rather than at the isoform level. Therefore, the specific contributions of Nogo-A, Nogo-B, and Nogo-C cannot be determined from the present data, and future isoform-specific validation is required.

Consistent with this possibility, our estimated immune cell composition analyses showed that *RTN4* expression was positively associated with monocytes and other myeloid cell subsets in both COPD and CAD cohorts. To further explore cell-type-specific expression patterns, we analyzed scRNA-seq data from PBMCs of CAD patients stratified by plaque rupture status. This analysis suggested that *RTN4*-associated signals were mainly localized to monocytes, particularly intermediate monocytes. Importantly, these single-cell findings should be interpreted as CAD-focused cellular context rather than direct evidence of *RTN4*-associated monocyte alterations in COPD–CAD comorbidity. Human blood monocytes can be broadly classified into classical CD14^+^CD16^−^, intermediate CD14^+^CD16^+^, and non-classical CD14^dim^CD16^+^ subsets [51,52,53]. Among them, intermediate monocytes are generally regarded as a transitional population and are characterized by phagocytic and immunoregulatory properties, together with relatively high intracellular levels of IL-1β and TNF-α under steady-state conditions [54,55,56]. Moreover, intermediate monocytes have been linked to cardiovascular risk and have been reported to independently predict cardiovascular events in patients undergoing elective coronary angiography [57]. In our single-cell analysis, *RTN4* expression was significantly higher in intermediate monocytes from PR samples than from NPR samples, whereas monocyte subset proportions were largely comparable between groups. These findings suggest that *RTN4*-associated differences may reflect functional changes in monocytes rather than major shifts in monocyte abundance.

This interpretation is consistent with evidence that monocyte activation is closely coupled to immunometabolic reprogramming during chronic inflammatory states [58,59,60,61]. Functional enrichment analyses and metabolic pathway scoring showed that *RTN4*-high intermediate monocytes exhibited transcriptional features related to leukocyte activation, cell adhesion, antigen presentation, oxidative stress responses, and metabolic remodeling. In particular, increased pentose phosphate pathway, glutathione metabolism, and xenobiotic metabolism activity may indicate enhanced demand for NADPH production, antioxidant defense, and redox adaptation [62,63]. Previous studies have shown that coordinated immune activation and redox-related metabolic remodeling in myeloid cells are associated with sustained inflammation and plaque vulnerability in cardiovascular disease [64]. Together, these findings suggest that *RTN4* upregulation may be associated with an intermediate monocyte immunometabolic state related to inflammatory amplification and plaque instability. Importantly, these analyses establish association rather than a functional role of *RTN4* in regulating monocyte activation or metabolism. *RTN4* knockdown or overexpression in monocytes, coupled with inflammatory and metabolic assays, will be required to establish causality.

Finally, our clinical validation further supported these transcriptomic findings. *RTN4* mRNA expression was increased in PBMCs from patients with COPD, with the highest levels observed in patients with COPD and comorbid CAD. Moreover, *RTN4* expression was negatively correlated with FEV1% predicted in the clinical cohort. Notably, *RTN4* expression was further increased in patients with COPD and comorbid CAD despite comparable lung function between the two COPD groups. This observation suggests that *RTN4* may capture CAD-related peripheral immune alterations beyond the degree of airflow limitation. Therefore, *RTN4* may serve as a PBMC-derived, monocyte-associated candidate molecular feature for identifying a COPD subgroup with cardiovascular comorbidity-related immune characteristics.

Several limitations should be acknowledged. First, although RT–qPCR validation supported the upregulation of *RTN4* in clinical PBMC samples, the cohort size was limited, and further validation in larger populations is needed. In addition, the clinical validation cohort did not include a CAD-only group, which limits our ability to determine whether the observed increase in *RTN4* expression is specifically associated with COPD–CAD comorbidity or primarily reflects CAD-related pathological changes. Future studies incorporating an independent CAD-only group, together with larger and prospectively characterized cohorts, will be important to clarify the disease specificity of *RTN4* expression. Although pulmonary function was comparable between the COPD and COPD+CAD groups, residual confounding related to COPD severity, smoking burden, exacerbation history, medication exposure, heterogeneity in CAD severity and cardiovascular risk factors such as hypertension, diabetes, and dyslipidemia cannot be fully excluded. Therefore, further validation in larger cohorts including healthy controls, COPD-only, CAD-only, and COPD+CAD groups, with comprehensive adjustment for relevant clinical factors, is needed to determine whether the association between *RTN4* expression and COPD–CAD comorbidity remains robust and whether *RTN4* represents a comorbidity-associated rather than disease-specific molecular feature. Second, the single-cell analysis provided useful cell-type-resolved evidence from CAD samples with different plaque rupture statuses; however, these samples were not derived from matched COPD–CAD cohorts. Given the clinical and technical challenges in obtaining matched single-cell datasets from patients with COPD and comorbid CAD, future studies using dedicated COPD–CAD single-cell cohorts are required to confirm the relevance of *RTN4*-high intermediate monocytes in this comorbidity context. In addition, the public transcriptomic cohorts differed in clinical characteristics and technical platforms, and residual cohort heterogeneity cannot be fully excluded despite the consistent direction of *RTN4* expression across independent datasets. Finally, the bulk transcriptomic datasets and the current RT–qPCR assay do not resolve individual *RTN4* isoforms, limiting isoform-specific biological interpretation. Future functional studies are warranted to further clarify the potential role of *RTN4* in monocyte activation and immunometabolic remodeling.

## 5. Conclusions

Overall, this study identified *RTN4* as a reproducible PBMC-derived, monocyte-associated molecular feature related to shared immunometabolic alterations in COPD and CAD. Clinical validation further supported *RTN4* upregulation in patients with COPD, particularly in those with comorbid CAD, while single-cell analysis suggested that *RTN4*-related changes were most evident in intermediate monocytes, especially in CAD samples with plaque rupture. These findings provide preliminary evidence supporting *RTN4* as a PBMC-derived, monocyte-associated molecular feature related to COPD–CAD comorbidity.

## Data Availability

The bulk RNA-seq datasets used in this study were retrieved from the Gene Expression Omnibus (GEO) database (https://www.ncbi.nlm.nih.gov/geo/) (accessed on 5 August 2025) under accession numbers GSE42057, GSE113079, GSE54837, and GSE250283. The single-cell RNA-seq dataset analyzed in this study was also obtained from GEO under accession number GSE269269 (https://www.ncbi.nlm.nih.gov/geo/) (accessed on 5 August 2025). The original anonymized clinical data and RT–qPCR data generated in this study are available from the corresponding author upon reasonable request, subject to ethical and institutional regulations.

## Supplementary Materials

The following supporting information can be downloaded at: https://www.mdpi.com/article/10.3390/genes17091046/s1, Figure S1. Estimated immune cell composition profiles in COPD and CAD cohorts. Stacked bar plots show the relative proportions of 22 immune cell types estimated by CIBERSORT in peripheral blood samples from the GSE42057 (COPD vs. control) and GSE113079 (CAD vs. control) datasets. Each bar represents an individual sample, and colors indicate different immune cell subsets. Tables S1–S12 are provided in a single supplementary excel file, with each table presented in a separate worksheet. Table S1. Gene information for WGCNA modules identified in the GSE42057 COPD cohort. Table S2. Gene information for WGCNA modules identified in the GSE113079 CAD cohort. Table S3. Results of Gene Ontology (GO) enrichment analysis of the overlapping genes. Table S4. Results of Kyoto Encyclopedia of Genes and Genomes (KEGG) pathway enrichment analysis of the overlapping genes. Table S5. Performance of machine-learning models in the GSE42057 COPD cohort. Table S6. Performance of machine-learning models in the GSE113079 CAD cohort. Table S7. Immune cell infiltration analysis of the GSE42057 cohort and correlations between immune cell proportions and RTN4 expression. Table S8. Immune cell infiltration analysis of the GSE113079 cohort and correlations between immune cell proportions and RTN4 expression. Table S9. Results of Gene Ontology (GO) enrichment analysis of genes upregulated in RTN4-high intermediate monocytes. Table S10. Scaled mean AUCell scores for selected metabolic pathways across NPR and PR groups stratified by RTN4 expression. Table S11. Primer sequences used for quantitative real-time PCR. Table S12. Characteristics and preprocessing of the public transcriptomic datasets included in this study.

## Abbreviations

COPD, chronic obstructive pulmonary disease; CAD, coronary artery disease; PBMCs, peripheral blood mononuclear cells; NK cells, natural killer cells; *RTN4*, reticulon-4; FEV_1_, forced expiratory volume in one second; FVC, forced vital capacity; WGCNA, weighted gene co-expression network analysis; PPI, protein–protein interaction; GEO, Gene Expression Omnibus; RT–qPCR, reverse transcription quantitative polymerase chain reaction; DM, diabetes mellitus; scRNA-seq, single-cell RNA sequencing; ACS, acute coronary syndrome; PR, plaque rupture; UMIs, unique molecular identifiers; PCA, principal component analysis; t-SNE, t-distributed stochastic neighbor embedding; GS, gene significance; MM, module membership; STRING, Search Tool for the Retrieval of Interacting Genes/Proteins; GO, Gene Ontology; KEGG, Kyoto Encyclopedia of Genes and Genomes; RF, random forest; SVM, support vector machine; XGBoost, extreme gradient boosting; GLM, generalized linear model; RMSE, root mean square error; ROC, receiver operating characteristic curve; AUC, area under the curve; *MAML3*, mastermind-like transcriptional coactivator 3; BMI, body mass index; ER, endoplasmic reticulum; MAMs, mitochondria-associated membranes; NADPH, reduced nicotinamide adenine dinucleotide phosphate; GOLD, Global Initiative for Chronic Obstructive Lung Disease; BMI, body mass index; IQR, interquartile range.
